# Immune-Enhancing Effects of *Lactobacillus plantarum* 200655 Isolated from Korean Kimchi in a Cyclophosphamide-Induced Immunocompromised Mouse Model

**DOI:** 10.4014/jmb.2103.03028

**Published:** 2021-04-05

**Authors:** Kyeong Jin Kim, Hyun-Dong Paik, Ji Yeon Kim

**Affiliations:** 1 Department of Nano Bio engineering, Seoul National University of Science and Technology, Seoul 01811, Republic of Korea; 2 Department of Food Science and Biotechnology of Animal Resources, Konkuk University, Seoul 05029, Republic of Korea; 3 Department of Food Science and Technology, Seoul National University of Science and Technology, Seoul 01811, Republic of Korea

**Keywords:** Cyclophosphamide, splenocytes, *Lactobacillus plantarum*, immune enhancement, MAPK/ERK signaling pathway

## Abstract

In this study, we evaluated the immune-enhancing activity of kimchi-derived *Lactobacillus plantarum* 200655 on immune suppression by cyclophosphamide (CP) in ICR mice. Animals were fed distilled water or 1×10^9^ colony-forming unit/kg B.W. 200655 or *Lactobacillus rhamnosus* GG as a positive control for 14 days. An in vivo model of immunosuppression was induced using CP 150 and 100 mg/kg B.W. at 7 and 10 days, respectively. Body weight, spleen index, spleen weight, and gene expression were measured to estimate the immune-enhancing effects. The dead 200655 (D-200655) group showed an increased spleen weight compared to the sham control (SC) group. Similarly, the spleen index was significantly higher than that in the CP-treated group. The live 200655 (L-200655) group showed an increased mRNA expression of tumor necrosis factor-alpha (TNF-α) and interleukin (IL)-6 in splenocytes. Also, the iNOS and COX-2 mRNA expression was upregulated in the L-200655 group compared to the CP-only (SC) group. The phosphorylation of ERK and MAPK was also upmodulated in the L-200655 group. These results indicate that *L. plantarum* 200655 ameliorated CP-induced immune suppression, suggesting that *L. plantarum* 200655 may have the potential to enhance the immune system.

## Introduction

The immune system plays a crucial defense-mechanism role by preventing pathogens and various infectious agents from initiating disease [1]. Homeostasis in the immune system is maintained by immunostimulation and immunosuppression, leading to immune regulation [2]. This defense system classically comprises the two sectors of innate and adaptive immunity [3]. In the non-specific defenses, known as innate immunity, neutrophils, macrophages, natural killer (NK) cells, and dendritic cells perceive exogenous factors and promote the production of cytokines [4]. When macrophages are activated, they eliminate the hazard directly by phagocytosis and indirectly by releasing inflammatory cytokines such as nitric oxide (NO), prostaglandin E_2_ (PGE_2_), tumor necrosis factor-α (TNF-α), and the interleukins IL-1β and IL-6 [5]. In contrast, the specific defenses, known as adaptive immunity, are characterized by T and B cells [6]. Inflammatory cytokines are also accompanied by the NF-κB and MAPK signaling pathways [7], which are mediated by toll-like receptors (TLRs) [8]. Physiological and pathological responses, including stress responses, inflammation, and apoptosis, are mediated by the MAPK signaling pathway in mammalian cells and tissues [9]. In the MAPK signaling pathway, phosphorylation of p38 MAPK and ERK1/2 plays important roles, activating the production of TNF-α, IL‐6, and IL‐8, which are inflammatory factors [10]. Moreover, the MAPK cascade and IL-8 secretion are triggered by TNF-α [11]. In addition to MAPK, NF‐κB regulates the expression of proinflammatory mediators. NF-κB p65 protein combines with IκB protein and exists in the cytoplasm. Depending on external stimuli, NF‐κB kinases (IKKs) can phosphorylate the IκB family of proteins, followed by ubiquitination and degradation, further phosphorylating p65 and releasing it into the nucleus, where it promotes inflammation.

Cyclophosphamide (CP) is a widely used alkylating cytotoxic agent that exerts broad-spectrum activities in modulating immune responses against various illnesses [1, 12]. However, administration of high-dose CP can cause immunosuppression and intestinal complications [13]. CP-induced immunosuppression decreases the number of lymphocytes in the blood and bone marrow, which results in inhibition of cytokine release from T cells [14].

Recently, it has become popular for immunocompromised people to ingest foods containing probiotics to enhance their immune systems [15]. Lactic acid bacteria (LAB) are gram-positive, non-spore forming, non-respiring cocci or rods that ferment yogurt, kimchi, and cheese [16]. *Lactobacillus* species, which can regulate the immune system and strengthen host defense against pathogens, are the main LAB in Korean kimchi, a traditional Korean fermented vegetable [17]. Among lactobacilli, *Lactobacillus plantarum* is a probiotic with immunomodulatory effects that can mitigate chronic intestinal inflammation and irritable bowel syndrome [18]. *Lactobacillus* species also promote the expression of IL-12 and interferon (IFN)-γ in monocytes and induce their differentiation into type 1 helper (TH1) T cell subsets [19]. In addition, previous studies reported that live *L. plantarum* HY7712 isolated from kimchi reverses the inhibition of NK and T cell activities caused by CP and c-irradiation in mice [16, 20].

The development of LAB with immune-improving properties is helpful for immunosuppressed people because of the lack of side effects. Recently, the inactivated form, as well as the live type, has been found to be beneficial for human health [21]. Although the mechanism is still unclear, many studies have reported that heat-killed LAB could show immunomodulatory activities. Likewise, studies have indicated the immune-enhancing effect of *L. plantarum* derived from kimchi on immunosuppressed mice, but the underlying mechanism remains poorly understood. The beneficial effects of probiotics depend on their species- and strain-specific characteristics [22]. Therefore, the immune-enhancing activity of *L. plantarum* 200655 must be investigated. The aim of this study was to elucidate the immuno-enhancing potential of live and dead LAB in immunosuppression of CP-treated mice with a focus on the MAPK signaling pathway.

## Materials and Methods

### Materials

Roswell Park Memorial Institute (RPMI) 1640 medium was purchased from Gibco-Life Technologies (USA); fetal bovine serum (FBS) and penicillin-streptomycin mixture from Biowest (France). Cyclophosphamide, lipopolysaccharide (LPS), and trypan blue were obtained from Sigma-Aldrich (USA).

### Preparation and Identification of LAB Strains

*L. plantarum* 200655 was provided by Konkuk University (Korea) and prepared as follows [23]: *L. plantarum* 200655 was isolated from cabbage kimchi. The kimchi sample (1 g) was serially diluted and inoculated on de Man, Rogosa, and Sharpe (MRS) agar at 37°C for 24 h. A colony was inoculated and incubated in MRS broth under the same condition. The commercial probiotic strain *L. rhamnosus* GG was obtained from the Korean Collection for Type Cultures (KCTC, Korea) and prepared as follows: LAB strains were incubated in MRS broth at 37°C for 18 h before use. To harvest the LAB cells, bacterial cell cultures were centrifuged at 14,240 ×*g* at 4°C for 10 min. The bacterial cells were washed thrice and resuspended in phosphate-buffered saline (Gibco, USA). The dead LAB was prepared by heating the harvested cells at 100°C for 1 h. *L. plantarum* 200655 was identified by 16S rRNA gene conducted by Bionics Inc. (Korea). The sequencing results were analyzed by comparison with the GenBank database using the Basic Local Alignment Search Tool (BLAST) website (http://blast.ncbi.nlm.nih.gov).

### Cyclophosphamide-Induced Immunosuppression Mouse Model

Seven-week-old ICR mice were purchased from Orient Bio (Korea) and maintained under a regular 12-h light/ 12-h dark cycle at 21–24°C, with relative humidity of 45%–60%, and a commercial diet. After 1 week of acclimation, the mice were randomly divided into five groups (n = 10) as follows: normal control (NC), CP only (SC), CP + 1 × 10^9^ CFU/kg B.W. *L. rhamnosus* GG as positive control (PC), CP + 1 × 10^9^ CFU/kg B.W. live *L. plantarum* 200655 (L-200655), and CP + 1 × 10^9^ CFU/kg B.W. dead *L. plantarum* 200655 (D-200655). The NC and SC groups were administered distilled water via oral gavage. Forty mice were injected intraperitoneally (i.p.) with CP in sterile saline (150 and 100 mg/kg) for 7 and 10 days to establish immunosuppression models. The experimental procedures (P204004) were approved by the Institutional Animal Care and Use Committee (IACUC) of KPC laboratory (Non-clinical contract research organization, Korea).

### NK Cell Activity Analysis

On day 14, whole blood from all groups was collected and gently mixed with NK cell-activating reagent, and the mixtures were then incubated for 24 h at 37°C. After incubation, each sample was centrifuged for 15 min. The supernatants were then carefully transferred to new tubes. Each supernatant of NK cell activity was measured by determining the levels of IFN-γ production using the Murine NK Activity Kit (ATGen, Korea) according to the manufacturer’s instructions.

### Alanine Aminotransferase (ALT) Activity in Serum

Serum ALT activity was determined to assess hepatocellular injury. ALT activity was measured using a colorimetric assay kit (Sigma Chemical) according to the manufacturer’s instructions. Briefly, the samples were centrifuged and stored in a freezer at -80°C until analysis. The enzyme activities of ALT in the sera were estimated using a microplate reader (BioTek, USA).

### Isolation of Splenocytes and Cell Culture

The spleens were removed to obtain splenocytes from mice. Next, the spleens were weighed and the spleen index was calculated by the following equation: spleen index (%) = [(spleen weight/body weight)] × 100. The spleens were then gently pressed with a syringe and forced through a 70-μm cell strainer (SPL Life Sciences, Korea). Cells were treated with red blood cell lysis buffer (Sigma-Aldrich), and isolated splenocytes were incubated in RPMI-1640 containing 10% (FBS) and 1% antibiotics (penicillin and streptomycin) with LPS in a 5%CO_2_ incubator.

### RNA Extraction and Quantitative RT-PCR

Total RNA was isolated from splenocytes using TRIzol reagent (Life Technologies, USA) following the manufacturer’s protocol. Total RNA was then reverse-transcribed using a high-capacity cDNA reverse transcription kit. The Universal Probe Library (UPL) method was used to quantify the expression of TNF-α, IL-6, IL-1β, and GAPDH in splenocytes using a Light Cycler 96 System (Hoffmann La Roche, Switzerland). The relative mRNA expression, normalized to GAPDH levels, was calculated using the comparative CT method. The sequences of the sense and antisense primers used for RT-qPCR analysis were as follows: TNF-α (forward, 5´-tcttctcattcctgcttgtgg-3´; reverse, 5´-ggtctgggccatagaactga-3´); IL-6 (forward, 5´-gctaccaaactggatataatcagga-3´; reverse, 5´-ccaggtagctatggtactccagaa-3´); IL-1β (forward, 5´-agttgacggaccccaaaag-3´; reverse, 5´- agctggatgctctcatcagg-3´); and GAPDH (forward, 5´-aagagggatgctgcccttac-3´; reverse, 5´-ccattttgtctacgggacga-3´).

### Protein Extraction and Western Blot Analysis

The cultured splenocytes were lysed by incubation for 20 min on ice in PRO-PREP Protein Extraction Solution (iNtRON Biotechnology, Korea). Protein was added to 2× sample buffer (Bio-Rad, USA), incubated at 90°C for 2 min, and electrophoresed on 10% sodium dodecyl sulfate (SDS) polyacrylamide gels. The resolved proteins in electrophoretic SDS polyacrylamide gel were transferred to a polyvinylidene difluoride (PVDF) membrane (Bio-Rad). The membrane was blocked with 5% skim milk for 1 h. The primary antibodies against ERK, phosphorylated ERK (p-ERK), p38 MAPK, and phosphorylated p38 MAPK (Cell Signaling Technology, USA) were added, and the membrane was incubated overnight at 4°C. After washing the membrane three times with TBST buffer (Tris-buffered saline, 0.1% Tween 20) for 5 min, the secondary antibody (Cell Signaling) was added, allowed to attach at room temperature for 2 h, and washed three times for 5 min with TBST buffer. The immunoreactive protein bands were developed with the ClarityTM Western ECL substrate (Bio-Rad) and quantified using a ChemiDoc MP Imaging System, which was followed by analysis using Image Lab software (Bio-Rad).

### Statistical Analysis

Data are expressed as the mean ± standard error of the mean (SEM). Statistical analyses were performed using Duncan’s multiple range test, which is a one-way analysis of variance (ANOVA) (SAS 9.4, SAS Institute, USA). Statistical significance was set at *p* < 0.05.

## Results

### Effect of 200655 on the CP-Induced Mouse Model

**Fig. 1:**
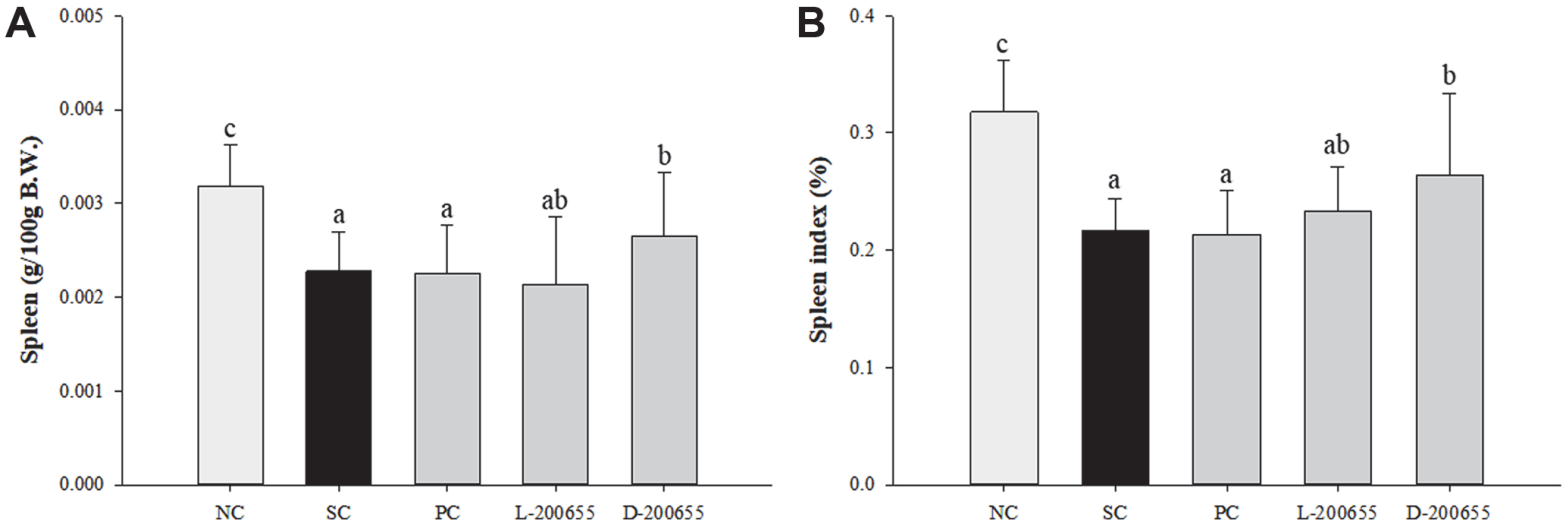
Regulatory effect of 12204 on spleen weight (A) and index (B) of CP-induced immunosuppressed mice. NC, normal control; SC, sham control; PC, positive control; L-200655, live 200655 lactic acid bacteria; D-200655, dead 200655 lactic acid bacteria. Values with different letters within a row are significantly different at *p* < 0.05, as determined by Duncan’s multiple range test. Values from small to large are arranged in alphabetical order.

To investigate the immune-enhancing effect of 200655 under physiological conditions, an immunosuppressed mouse model was induced by intraperitoneal administration of CP. The effects of each LAB strain on body and spleen weight and spleen index are shown in Table 1 and Fig. 1, respectively. There was no significant difference in the body weight of all groups on day 0, on which oral administration of each LAB strain was commenced. On day 7, body weight increased in all groups. Because CP was injected, body weight decreased in all groups except for the NC group. In the SC group, body weight was significantly reduced by approximately 4.9% compared to the NC group on day 14. The body weights of the PC, L-200655, and D-200655 groups decreased by approximately 5.1%, 2.2%, and 7.5%, respectively, compared to the SC group. The highest loss in body weight was observed in the D-200655 group. Similarly, the NC group showed the highest spleen weight and index. In contrast, the reduction in spleen weight caused by CP significantly improved in mice administered D-200655. Likewise, the spleen index was significantly increased in the D-200655 group compared with that in the SC group.

### Whole Blood NK Activity and Serum ALT Levels in the CP-Induced Mouse Model

As shown in Table 2, IFN-γ levels from NK cells were decreased in SC group (163.10 ± 20.55 pg/ml) compared to NC group (212.97 ± 36.98 pg/ml). However, the suppression of NK cell activity by CP was recovered by treatment with 200655 (L-200655, 170.09 ± 19.76 pg/ml; D-200655, 175.64 ± 12.44 pg/ml), but there was no significant difference. The effects of 200655 on CP-induced elevation of serum ALT activity are shown in Table 2. Increased ALT activity by CP in all sample groups was alleviated compared with the SC group, but the difference was not significant.

### Effect of 200655 on Inflammatory Cytokine Expression in Splenocytes

**Fig. 2:**
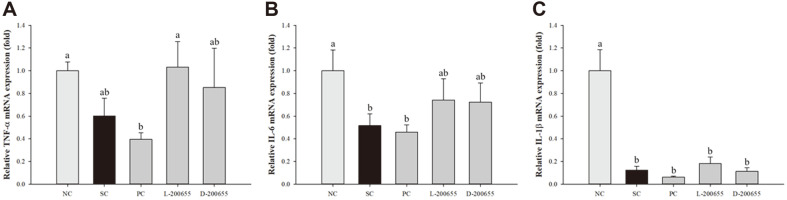
Relative mRNA expression profile of proinflammatory cytokines in splenocytes treated with LPS as mitogen in CP-induced immunosuppressed mice. (**A**) TNF-α; (**B**) IL-6; (**C**) IL-1β. NC, normal control; SC, sham control; PC, positive control; L-200655, live 200655 lactic acid bacteria; D-200655, dead 200655 lactic acid bacteria. Values with different letters within a row are significantly different at *p* < 0.05, as determined by Duncan’s multiple range test. Values from small to large are arranged in alphabetical order.

To test the immune-improving effect of 200655 on splenocytes, RT-qPCR was conducted for all experimental groups. The outcomes reconfigured the case in which SC significantly reduced the levels of inflammatory mRNA expression, namely, TNF-α, IL-6, and IL-1β (Fig. 2). In splenocytes from the SC group, the expression of proinflammatory cytokines sharply decreased by approximately 40%, 48%, and 88% in comparison to that in the NC group. In contrast, the L-200655 and D-200655 groups showed higher relative mRNA expression of TNF-α by about 1.7- and 1.4-fold, respectively, compared to the SC group. Similarly, relative mRNA expression of IL-6 was recovered by approximately 1.4- and 1.4-fold in the L-200655 and D-200655 groups, respectively, compared to the SC group. Although L-200655 showed an increased tendency of IL-1β mRNA expression, there was no significant difference in all groups except for the NC group. The mRNA expression of these cytokines was almost recovered in L-200655 compared to D-200655.

### Activation of the MAPK Signaling Pathway

**Fig. 3:**
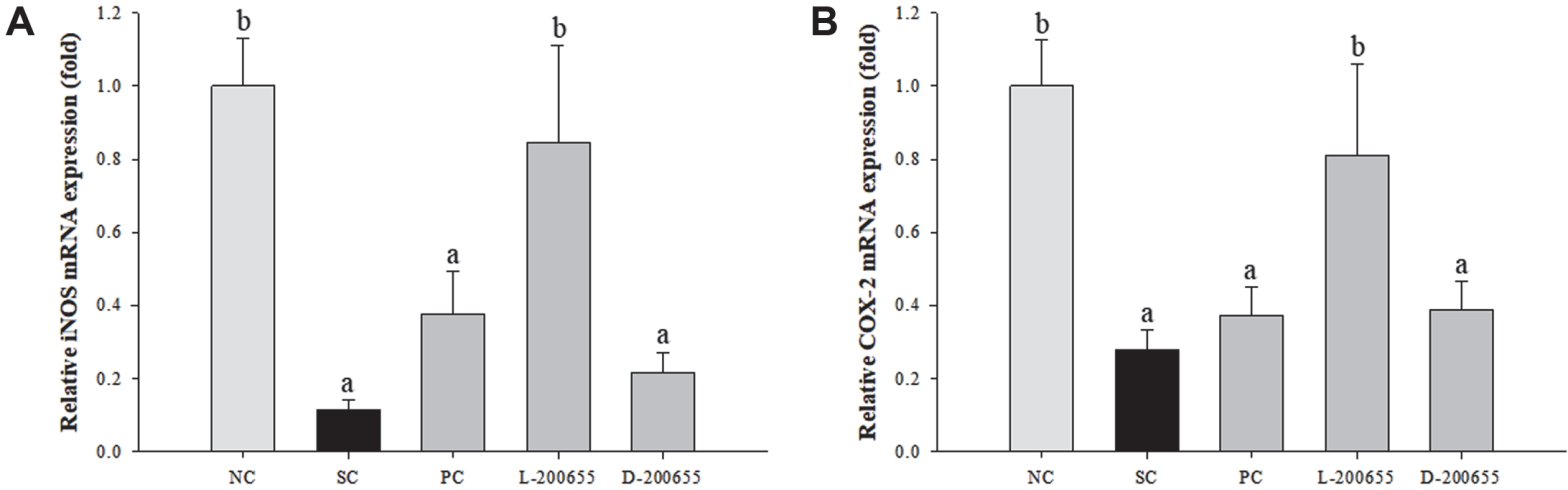
Relative mRNA expression of proinflammatory mediators in splenocytes treated with LPS as mitogen in CP-induced immunosuppressed mice. (**A**) iNOS; (**B**) COX-2; NC, normal control; SC, sham control; PC, positive control; L-200655, live 200655 lactic acid bacteria; D-200655, dead 200655 lactic acid bacteria. Values with different letters within a row are significantly different at *p* < 0.05, as determined by Duncan’s multiple range test. Values from small to large are arranged in alphabetical order.

**Fig. 4:**
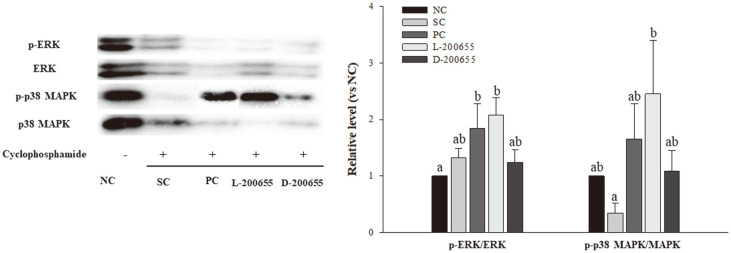
Effect of 200655 on MAPK signaling pathway in splenocytes treated with LPS as mitogen in CP-induced immunosuppressed mice. NC, normal control; SC, sham control; PC, positive control; L-200655, live 200655 lactic acid bacteria; D-200655, dead 200655 lactic acid bacteria. Values with different letters within a row are significantly different at *p* < 0.05, as determined by Duncan’s multiple range test. Values from small to large are arranged in alphabetical order.

In addition to the inflammatory cytokine levels, iNOS and COX-2 mRNA levels were confirmed as immune-enhancing responses. In the SC group, the iNOS and COX-2 mRNA levels decreased by approximately 89% and 72%, respectively, compared to the NC group (Fig. 3). In contrast, each of the three LAB groups displayed increased relative mRNA expression of iNOS by approximately 3.3-, 7.4-, and 1.9-fold in the PC, L-200655, and D-200655 groups, respectively, compared to the SC group. However, there was no significant difference between the PC and D-200655 groups. Likewise, the relative mRNA expression of COX-2 was increased by approximately 1.3-, 2.9-, and 1.4-fold in the PC, L-200655, and D-200655 groups, respectively, compared to the SC group. However, there was no significant difference between the PC and D-200655 groups. The phosphorylation of ERK was upregulated by approximately 1.3-fold in the SC group compared to the NC group (Fig. 4). After administration of LAB, the phosphorylation of ERK was also observed, except for the D-200655 group, by approximately 1.3- and 1.6-fold in the PC and L-200655 groups, respectively, compared to the SC group. In terms of p38 MAPK, the SC group showed decreased phosphorylation of p38 MAPK by approximately 65% compared to the NC group. Meanwhile, each of the three LAB groups was upregulated by approximately 4.7-, 7-, and 3.1-fold in the PC, L-200655, and D-200655 groups, respectively, compared to the SC group.

## Discussion

Various studies have shown that bioactive compounds promote immune responses when the body’s immune function is degraded owing to infection or disease. In accordance with emerging issues of biological stability for chemotherapy and immuno-disease treatment, which have side effects, harmless substances such as plants, fruits, and LAB have been studied as immune regulators as well as for their health-promoting properties. Therefore, this study was performed to verify and support the hypothesis that LAB derived from kimchi could modulate activation of the MAPK signaling pathway in a mouse model featuring deteriorated immunity.

CP is a well-known immunosuppressive drug used in clinical research that damages DNA structure, kills immune cells, interferes with proliferation and differentiation of macrophages of T and B cells, and restrains the humoral and cellular immune responses [24]. In the long term, high-dose CP treatment triggers immunosuppression accompanied by a reduction in body weight, relative weight of the spleen and thymus, immune system factors, lymphocyte proliferation, and NK cell activity [25]. In this study, the changes in body weight and spleen were confirmed by CP. Compared with the NC group, the SC group that received CP showed a significant decrease in body weight. Similarly, each LAB-treated group showed a noticeable reduction in weight. These results suggest that the injection of CP had a greater influence on apoptosis in spleen cells than LAB had on cell proliferation [26]. As a negative influence, spleen weight was significantly lower in the SC group than in the NC group. In the same vein, the spleen index also decreased in the SC group. However, both the L-200655 and D-200655 groups significantly inhibited the reduction of spleen weight and index. They can therefore have a positive effect on the immune system. CP challenge also induces liver damage, which was revealed by increased levels of ALT and AST in the serum [27, 28]. The SC group showed an increase in ALT concentration compared to that in the NC group. These results suggest that CP treatment has considerable hepatotoxic effects. In this study, although the PC and D-200655 groups showed low levels of ALT, there were no significant differences.

NK cells are involved in the first step of the reaction to infection and play an important role in innate immune responses [29, 30]. Major proinflammatory cytokines such as IFN-γ and TNF-α are released from NK cells and modulate immune responses in infected cells [31]. Previous study reported that patients with diseases have considerably reduced IFN-γ levels and do not properly regulate NK cell activity [32]. These findings could contribute to the relationship between NK cell activity and immunological disorders by measuring IFN-γ concentration in NK cells. The effects of CP and/or LABs on NK cell activity were confirmed using whole blood. Although there was no significant difference between the groups, both the L-200655 and D-200655 groups showed an increase in IFN-γ concentration compared with the SC group. These results suggest that *L. plantarum* 200655 derived from kimchi can potentially recover NK cell activity.

The spleen is a crucial immune lymphoid organ in the body, with resident T and B lymphocytes as well as monocytes and macrophages for persistence of immunological homeostasis [33, 34]. LPS is a mitogen that causes cellular proliferation in B lymphocytes [35]. In this study, splenocytes from each group were co-incubated with LPS. This resulted in an increase in proinflammatory cytokines (TNF-α and IL-6) and mediators (iNOS and COX-2) in both the L-200655 and D-200655 groups. Overall, the mRNA expression of TNF-α, IL-6, IL-1β, iNOS, and COX-2 was the highest in the L-200655 group. Although the MAPK signaling pathway was also activated in all groups compared with the SC group, L-200655 showed marked phosphorylation of both ERK and p38 MAPK. Notably, the SC group displayed increased phosphorylation of ERK, unlike p38 MAPK (Fig. 4). It could be considered that CP showed anti-tumor immunity through immune potentiation. A previous study described that the anti-tumor effects of CP depend on the dose administered and revealed immune potentiation or direct cytolytic activity [36]. Another study also reported that CP injection of 70 mg/mouse shows immune potentiation and expedites tumor growth in combination with an exosome-based vaccine against large established solid tumors [37]. Numerous intracellular signaling pathways are linked to inflammatory responses [38]. Among them, MAPK, including p38, ERK1/2, and JNK, regulate cell growth and development with cellular responses to cytokines and stresses [9], and MAPK pathways are accompanied by NF-κB for modulation of proinflammatory cytokines and inflammatory processes. In addition, many studies have reported that the regulation of both iNOS and COX-2 is associated with activation of the MAPK signaling pathway. In this study, the MAPK signaling pathway was an important molecular target of LAB derived from kimchi.

LAB exhibit multiple biological effects, including anti-obesity, anti-inflammatory, anti-staphylococcal, anti-allergic, immune-enhancing, and antioxidant activities. Muhammad *et al*. described that, because probiotic effects are strain-specific, they do not behave via the same mechanisms [39]. The mechanism underlying the biological activities should be investigated in each relevant strain because the related response pathways vary. In addition, further study to examine why different effects appear in live and heat-killed LAB needs to be performed. As a potential reason, it could be considered that denaturation of the cell membrane composition is caused by heating, which results in various effects on immune responses. Chu-Chun Ou *et al*. reported that heat-treated LAB leads to longer expiration date, easier storage, and more stable transportation of product [40]. This could be seen as contributing to applicability in the food industry. Although many studies have shown that LAB originated from kimchi could attenuate immunocompromised status, to our knowledge, this is the first study to show that *L. plantarum* 200655-derived kimchi improves immunosuppression through regulation of the p38 MAPK/ERK signaling pathway in a CP-induced immunosuppression mouse model. However, a limitation of our study is that detailed mechanisms related to the p38 MAPK/ERK signaling pathway at the molecular level could not be demonstrated. Therefore, further studies are required to elucidate the exact mechanisms.

## Figures and Tables

**Table 1:** Effect of 200655 on body weight in CP-induced immunosuppressed mice.

Group	Body weight (g)		

	Day 0	Day 7	Day 14
NC	36.10 ± 0.31	37.33 ± 0.54	34.73 ± 0.55^a^
SC	36.08 ± 0.29	37.34 ± 0.43	34.02 ± 0.41^ab^
PC	36.07 ± 0.27	36.20 ± 0.36	33.29 ± 0.39^ab^
L-200655	36.07 ± 0.27	36.87 ± 0.38	33.70 ± 0.44^ab^
D-200655	36.03 ± 0.25	36.64 ± 0.39	32.93 ± 0.64^b^

NC, normal control; SC, sham control; PC, positive control; L-200655, live 200655 lactic acid bacteria; D-200655, dead 200655 lactic acid bacteria. Values with different letters within a row are significantly different at *p* < 0.05, as determined by Duncan’s multiple range test. Values from small to large are arranged in alphabetical order.

**Table 2:** Effect of 200655 on NK cell and alanine aminotransferase activity in CP-induced immunosuppressed mice.

Group	IFN-γ (pg/ml)	ALT (mU/ml)
NC	212.97 ± 36.98	38.97 ± 18.97
SC	163.10 ± 20.55	40.75 ± 19.79
PC	154.11 ± 9.43	33.94 ± 10.66
L-200655	170.09 ± 19.76	39.29 ± 12.12
D-200655	175.64 ± 12.44	36.70 ± 15.09

NC, normal control; SC, sham control; PC, positive control; L-200655, live 200655 lactic acid bacteria; D-200655, dead 200655 lactic acid bacteria. Values with different letters within a row are significantly different at *p* < 0.05, as determined by Duncan’s multiple range test. Values from small to large are arranged in alphabetical order.
